# Minor Paragraphs

**Published:** 1886-04

**Authors:** 


### A LITTLE LEARNING IS A DANGEROUS THING.

The smart young lady who wrote a note to the doctor asking him to visit her brother, and bring his urethra with him, has been discounted by a well informed medical student of Indianapolis, who was asked recently by his sweetheart to examine her throat for some slight ailment. Being anxious to exhibit his embryonic medical talents to his fair inamorata, he called for a spoon, dextrously depressed her tongue, gazed knowingly into the yawning chasm thus brought into view, and then, with a look of profound wisdom, informed her that *her vulva was greatly elongated.—Indianapolis Medical Journal.*

---

### A BENEFACTION.

If he who makes two blades of grass grow where only one grew before is called a benefactor, what shall we call a practiced, scientific chemist who produces a staple drug of such virtue that it has *six times* the power and value of any antecedent preparation of the same drug? Surely *no less* a benefactor than the grass man. Well, W. F. Kidder & Co., of New York, have, after two years experimenting, succeeded in perfecting a non-hydroscopic pepsin which will be advertised in these pages next issue as "Kidder's Crust Pepsin." Of course, it is understood that the value of pepsin is in proportion to its albumen-solving power. This preparation dissolves 1000 grains of egg albumen by one grain of pepsin. To make the saccharated pepsin of the U. S. D. it requires only one grain of Kidder's Crust Pepsin to 18 grains sugar of milk; whereas by the standard process it takes six grains of pepsin; therefore the Crust pepsin has a value of six times that of the best pepsin now available; and being sold at the same price, it follows that Kidder & Co. have outdone the grass test, and now make one grain of pepsin do what ordinarily requires, in the physician's hands, six grains! And as it is *the* remedy *par excellence* for dissolving *diphtheritic membrane* (and even chancres), it follows that a physician can *cure six cases* of diphtheria with the means he formerly possessed of curing one case. If this is not a logical deduction, what is? We anticipate a tremendous demand for this new and powerful solvent.

### DEATH OF DR. FLINT.

The death of Prof. Anstin Flint, Sr., is peculiarly unfortunate just at this juncture, and will be a telling loss to the coming International Medical Congress. He was the most influential medical man in America—was to America what Virchow is to Germany, Charcot to France, and Gull, Hutchinson and Sir H. Thompson are to England, the acknowledged head of the profession, the American Watson. While he was undoubtedly the backbone of the International Medical Congress "as now proposed to be organized"(?) he was at the same time the great conservative element from which, if from any source, amicable adjustment was expected to come. That is to say, while all had respect for his opinion, there was none of that personal animosity towards him that undoubtedly so unjustly exists towards our great Davis; and Shoemaker. His death weakens our cause, but does not destroy the chances for a successful congress.

### DELEGATES.

Do not forget to come to Dallas prepared to pay your subscription to the JOURNAL; it is a matter of much importance to us, in the aggregate, though a trifle to each individual. We have sent the JOURNAL ten months to many physicians who have not yet paid for it, and this is to GIVE NOTICE that this is the *last number* which will be sent to those who have not paid up, or who don't pay prior to May 1st. The names of all such will be dropped at once. It costs money to run a journal, and we cannot afford to send it any longer for nothing. A word to the wise is sufficient. No physician should be without his home journal unless he wants to get left. Why, we had a letter recently enquiring' "when the State Medical Association would have another meeting?" The JOURNAL will keep you informed of everything that is going on in the medical world, and particularly what the home profession is doing.

MEMORIAL.

At a recent meeting of the Board of Regents of the University, Dr. A. G. Clopton, of Jefferson, Texas, was choosen to deliver the memorial address (on the life and services of the late Dr. Ashbel Smith), at the commencement of the University, which takes place June 16th.

---

Dr. T. D. Wooten has been elected president of the Board of Regents of the University of Texas, vice Ashbel Smith, deceased.

---

APOLOGETIC.

We have been compelled to give all our space usually occupied by "Original Communications" on strictly medical topics, as well as "Cullings of Clinical Matters from Exchanges," to the important and interesting biographical sketch of Dr. Ashbel Smith; hence there is less of strictly medical reading in this number than usual. The time for the annual medical conventions, too, being at hand, we have much to say of interest to delegates and others—under the heading "Society Notes,"—to which we have given more room than usual. Although this number of the JOURNAL contains less of strictly medical matters than customary, it will be found full of medical news and editorials on subjects in which all feel more or less interest.

